# A safety study of 500 μA cathodal transcranial direct current stimulation in rat

**DOI:** 10.1186/s12868-019-0523-7

**Published:** 2019-08-06

**Authors:** Keying Zhang, Ling Guo, Junping Zhang, Guangzhou An, Yan Zhou, Jiajin Lin, Junling Xing, Mai Lu, Guirong Ding

**Affiliations:** 1 Department of Radiation Protection Biology, Faculty of Preventive Medicine, Fourth Military Medical University, Xi’an, 710032 People’s Republic of China; 2 Military Health Team of 61255 Troops of the Chinese People’s Liberation Army, Houma, 043000 People’s Republic of China; 3 Key Lab. of Opt-Electronic Technology and Intelligent Control of Ministry of Education, Lanzhou Jiaotong University, Lanzhou, 730000 People’s Republic of China

**Keywords:** tDCS, Safety, Rat, Brain, Morphology

## Abstract

**Background:**

Transcranial direct current stimulation (tDCS) is a noninvasive neural control technology that has become a research hotspot. To facilitate further research of tDCS, the biosafety of 500 μA cathodal tDCS, a controversial parameter in rats was evaluated.

**Results:**

24 animals were randomly divided into two groups: a cathodal tDCS group (tDCS, n = 12) and control group (control, n = 12). Animals in the tDCS group received 5 consecutive days of cathodal tDCS (500 μA, 15 min, once per day) followed by a tDCS-free interval of 2 days and 5 additional days of stimulation, totally two treatments of tDCS for a total of 10 days. Computational 3D rat model was adopted to calculate the current density distributions in brain during tDCS treatment. Essential brain functions including motor function and learning and memory ability were evaluated. Additionally, to estimate the neurotoxicity of tDCS, the brain morphology, neurotransmitter levels and cerebral temperature were investigated. Our results showed that the current density inside the brain was less than 20 A/m^2^ during tDCS treatment in computational model. tDCS did not affect motor functions and learning and memory ability after tDCS treatment. In addition, no significant differences were found for the tDCS group in hematology, serum biochemical markers or the morphology of major organs. Moreover, tDCS treatment had no effect on the brain morphology, neural structures, neurotransmitter levels or cerebral temperature.

**Conclusion:**

500 μA cathodal tDCS as performed in the present study was safe for rodents.

**Electronic supplementary material:**

The online version of this article (10.1186/s12868-019-0523-7) contains supplementary material, which is available to authorized users.

## Background

Transcranial direct current stimulation (tDCS) is a noninvasive neural control technology that has been increasingly tested as a tool to modulate plasticity in neuropsychiatric diseases [1–4]. As reported recently, clinical tDCS trials induce beneficial effects in several neurological and psychiatric contexts including pain [5], motor rehabilitation [6], chronic stroke [7], cognitive function [8], major depression [9], and epilepsy [10]. Although tDCS has exhibited therapeutic potential and application prospect in clinical trials, its mechanism remains largely unclear.

To facilitate further research of tDCS, it is important to confirm the biosafety of tDCS. Furthermore, as tDCS paradigms employing increased intensities or prolonged stimulation durations are developed, it is necessary to update the safety thresholds. At present, in human studies, relatively large wet sponges (typically 25–35 cm^2^) are commonly used. Based on a total of more than 33,000 sessions in over 1000 subjects [11], there has been no evidence for irreversible injury produced by conventional tDCS protocols within a wide range of stimulation parameters (stimulation duration ≤ 40 min, current ≤ 4 mA and conventional charge ≤ 7.2 C). In rats, the safety of tDCS has been more controversial. Liebetanz and colleagues [12] reported that brain lesions were observed at a current strength of 500 μA in cathodal tDCS with a duration of 10 min, which corresponds to a charge density of 85,714 C/m^2^ based on the epicranial electrode area of 3.5 mm^2^. Charge density was commonly used as an important index for definition of tDCS safety threshold. If brain lesions were observed at a certain charge density, this charge density would be defined as the safety threshold. However, recent studies have shown that a current strength of 500 μA over a duration of 15 min were used in cathodal tDCS is safe for rats [13, 14], which corresponds to a charge density of 128,571 C/m^2^ based on the epicranial electrode area of 3.5 mm^2^. Accordingly, the safety threshold for tDCS charge density has been increased to at least 128,571 C/m^2^, despite the fact that Liebetanz and colleagues [12] consider these levels to be harmful to the rodent brain.

To confirm safety thresholds for tDCS, the biosafety of 500 μA cathodal tDCS was evaluated by observing behavioral changes and neurotoxicology after tDCS administration in rats. Additionally, considering that electricity can flow through the body during tDCS process, general animal health and non-brain organs were also evaluated.

## Results

### Currents distributions in computational rat model

**Fig. 1:**
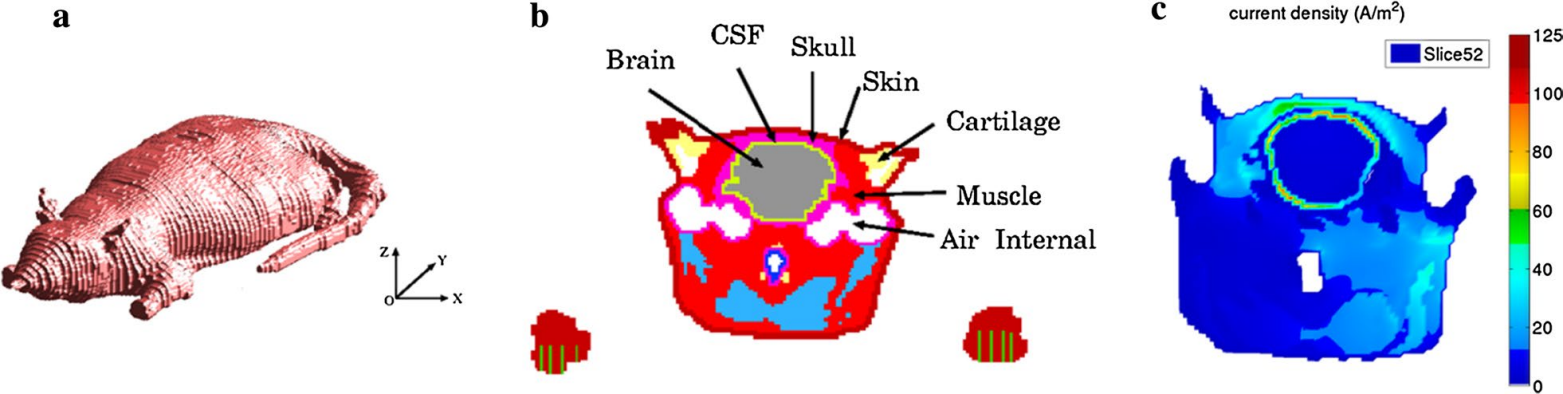
Computational rat model and current density distribution during tDCS. **a** 3D rat model. **b** Tissue distribution. **c** Current density distribution

As shown in Fig. 1a, the typical slice layers at the coordinates: Coronal plane, y = 52 mm, which contain the rat brain. The tissue distribution such as skin, brain, skull, fat and muscle were shown in Fig. 1b. The current density in rat model during tDCS treatment was shown in Fig. 1c and Additional file 1. As we can see, the current density varied from different rat tissues, and the current density inside the brain was less than 20 A/m^2^ and skull was 40–60 A/m^2^ in computational model.

### 500 μA cathodal tDCS had no obvious effects on motor function

**Fig. 2:**
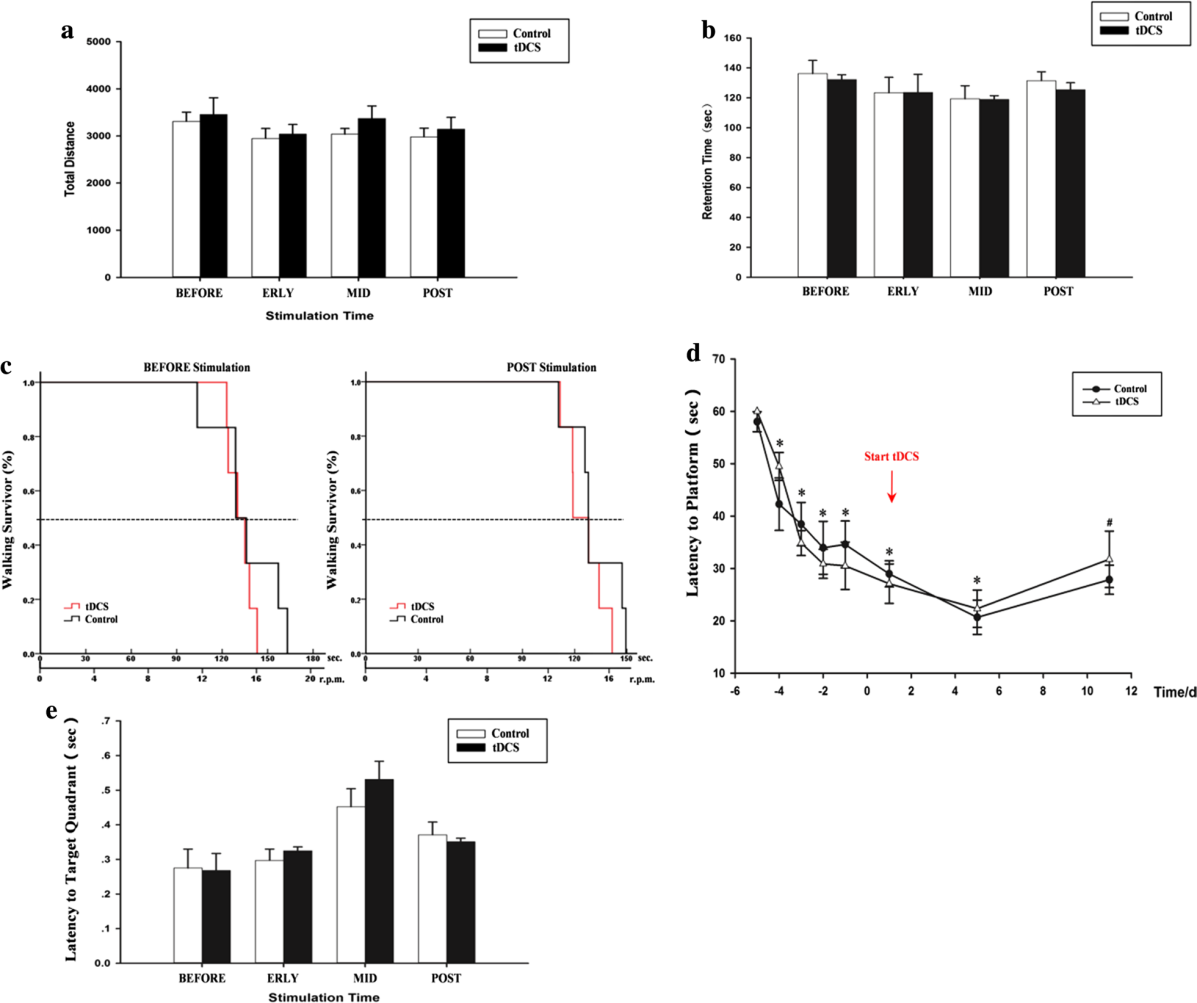
Effects of cathodal tDCS on brain functions. **a** Total distance traveled in the OFT. **b** Retention time on the rotarod. **c** Time-dependent changes in performance on the rotarod between BEFORE and POST phases. **d** Latency to the platform on the spatial acquisition test. **e** Durations in target quadrant on probe trials. Data were presented as the mean ± SEM. N = 6 rats per group. *p < 0.05 relative to the first acquisition day, ^#^*p* < 0.05 relative to the seventh acquisition day. No significant difference was observed between tDCS and control groups

Motor function did not differ between controls and the tDCS group on either the OFT (which assessed locomotor activity) or the rotarod test (which assessed athletic endurance). Relative to the control group, the tDCS group exhibited no obvious effects from epicranial electrode implantation on total distance traveled in the OFT at the BEFORE timepoint. Similarly, distance traveled did not change in the ERLY, MID and POST phases (Fig. 2a and Additional file 2). Moreover, retention time on the ratarod, a symbol of athletic endurance, exhibited no difference between these two groups (Fig. 2b, c and Additional file 3). In addition, no relationship was found between motor function and cumulative stimulation time. This null finding suggested that the bio-effect of cathodal tDCS did not have significant cumulative effects on animals’ motor behavior in present parameter.

### 500 μA cathodal tDCS had no discernible effects on learning and memory ability

Learning and memory, which are among the most important brain functions, were evaluated with the MWM. Learning was assessed during the spatial acquisition phases. Our data showed that escape latency to the hidden platform continued to decrease after the first trial in each session, with significant differences observed between the first acquisition day and days 2–7 (*p* < 0.05). This difference indicated that rats had learned the location of the platform on the second day. However, escape latency increased on the eighth acquisition day relative to the seventh (*p* < 0.05). This increase may be due to the inclusion of multi-probe trials, i.e., the sudden removal of the platform led to a disorder of spatial memory (Fig. 2d and Additional file 4). On probe trials, durations in the target quadrant, a symbol of reference memory, displayed a similar tendency to durations measured during the spatial acquisition phase (Fig. 2e and Additional file 5). However, no significant differences were found between the tDCS and control groups in either the learning or recall phase.

### 500 μA cathodal tDCS had no obvious effects on hematology and serum biochemical markers

**Fig. 3:**
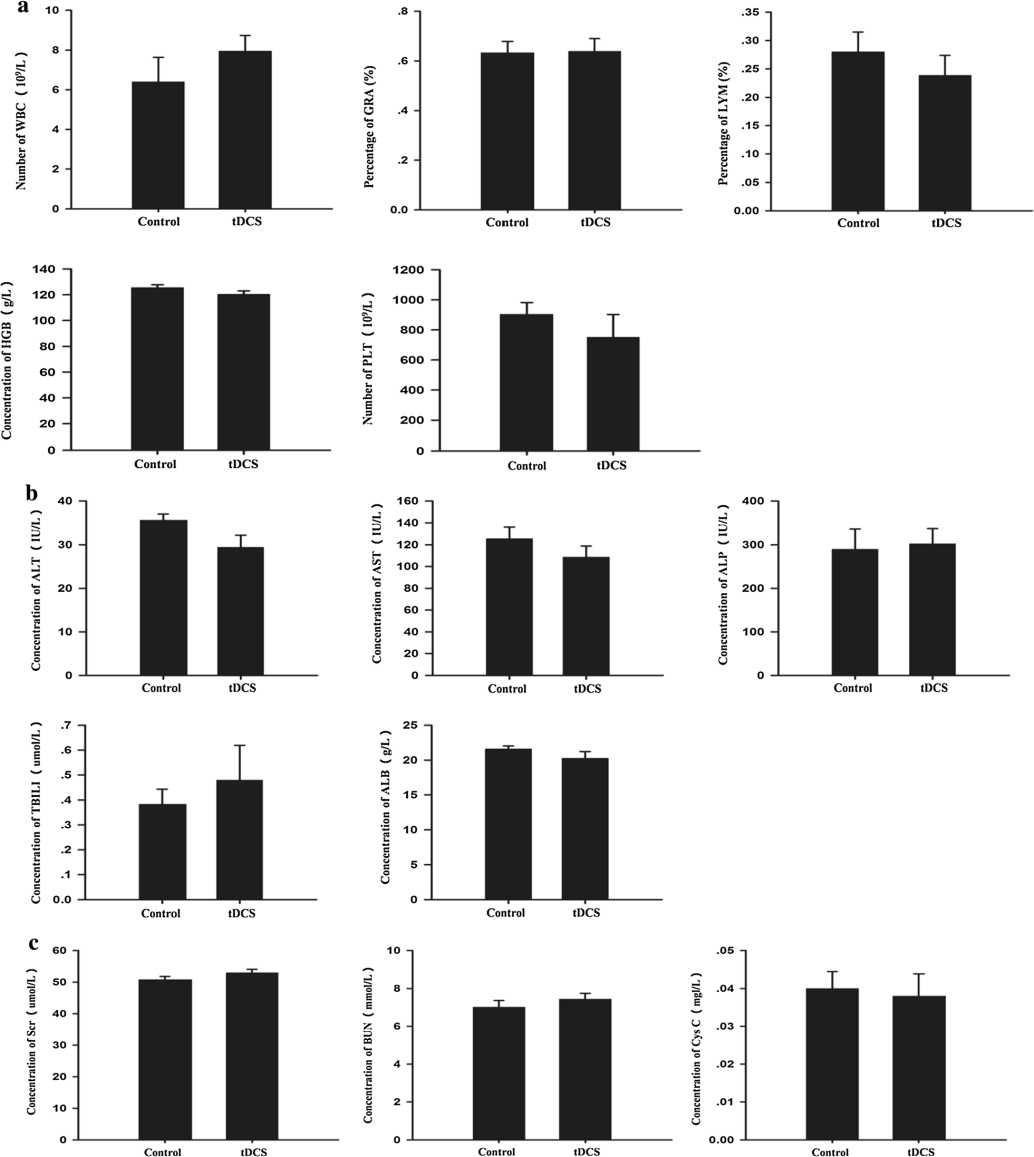
The results of hematology and serum biochemical marker quantification after cathodal tDCS. **a** Hematology results. **b** Levels of serum biochemical markers for liver function. **c** Levels for kidney function. Data were presented as the mean ± SEM. N = 6 rats per group. No significant difference was observed between tDCS and control groups

Hematology and serum biochemical markers are common physiological indexes that were used to evaluate the health of animals. The number of WBC and PLT, the percentage of LYM and GRA, and the concentration of HGB were assessed by hematology analysis. These indexes displayed no significant differences between the tDCS and control groups (Fig. 3a and Additional file 6). Concentrations of serum biochemical markers for liver function (Fig. 3b and Additional file 7) such as ALT, AST, ALP, TBILI and ALB exhibited no remarkable changes after tDCS treatment. A similar result was obtained for serum biochemical markers of kidney function (Fig. 3c and Additional file 8): Namely, concentrations of Scr, BUN and Cys C in the tDCS group did not significantly differ from those of the control group.

### 500 μA cathodal tDCS had no effect on the morphology of major organs

**Fig. 4:**
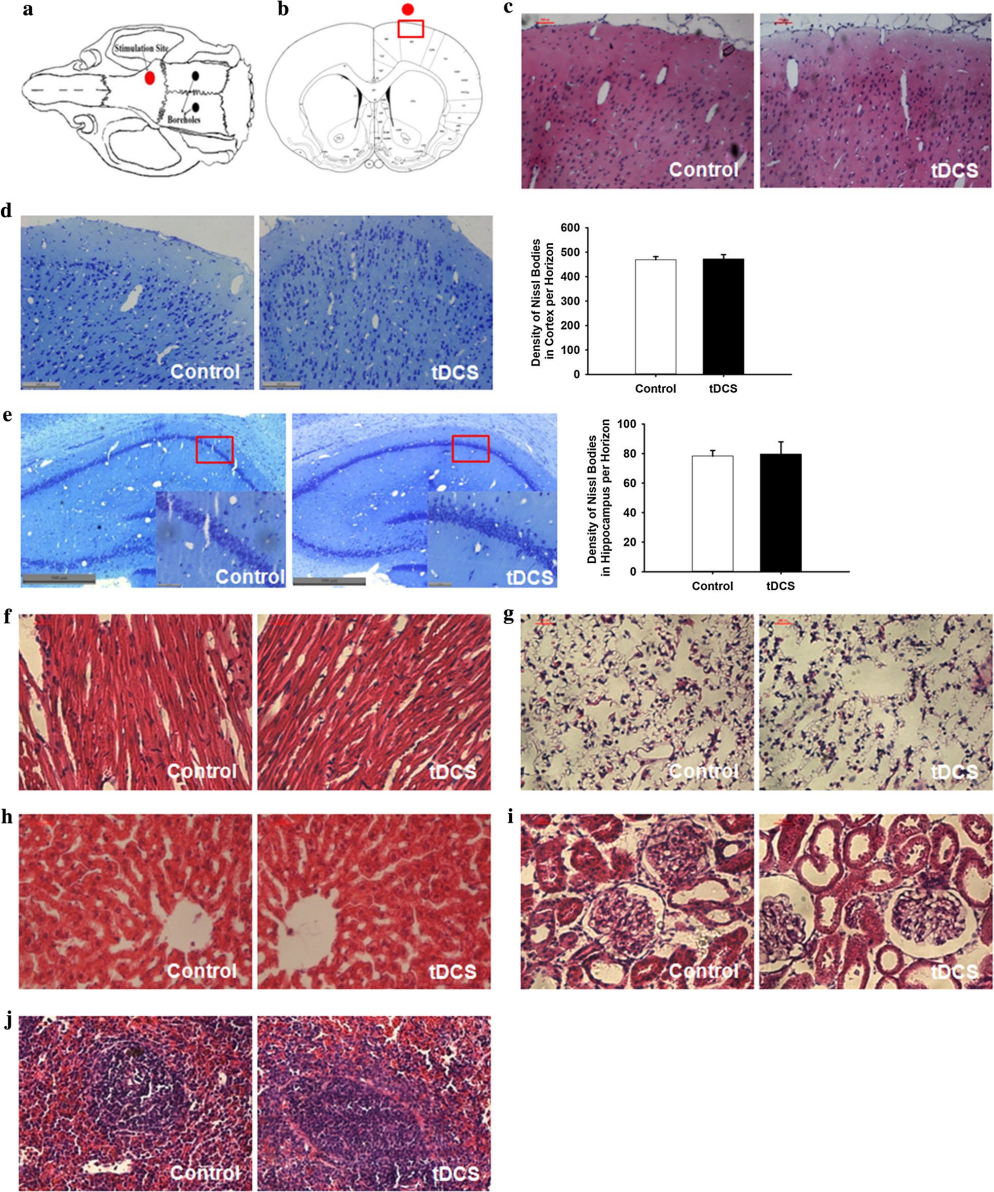
The morphology of major organs examined by HE staining and Nissl’s staining after cathodal tDCS. Coordinates of the tDCS site on the skull (**a**) and the epicranial electrode location (red circle, **b**) were schematically depicted. The morphology of cortex stained with HE (**c**) and neurons in cortex and hippocampus stained with Nissl’s staining (**d**, **e**). The morphology of heart (**f**), lung (**g**), liver (**h**), kidney (**i**) and spleen (**j**). Scale bar = 25 μm. Data were presented as the mean ± SEM. N = 6 rats per group. No significant difference was observed between tDCS and control groups

Histological examination of the brain, heart, lung, liver, kidney and spleen were performed to verify whether 500 μA of cathodal tDCS administration for 10 days resulted in morphological change. After 500 μA cathodal tDCS, histological analysis revealed that none of the rats had suffered from a cortical lesion (Fig. 4a–c). In addition, as one of neural characteristic structures, the Nissl bodies in brain were also observed in this study. It was found that the shape and density of intraneural Nissl bodies in cortex and hippocampus did not change compared with control group (Fig. 4d, e and Additional file 9), which was consistent with the results of the behavior assessment. What’s more, no epileptic seizures were observed during the stimulation period. Additionally, the morphology of other major organs did not obviously change after tDCS treatment compared with the control group, as shown in Fig. 4f–j.

### 500 μA cathodal tDCS had no effect on brain neurotransmitter levels

**Fig. 5:**
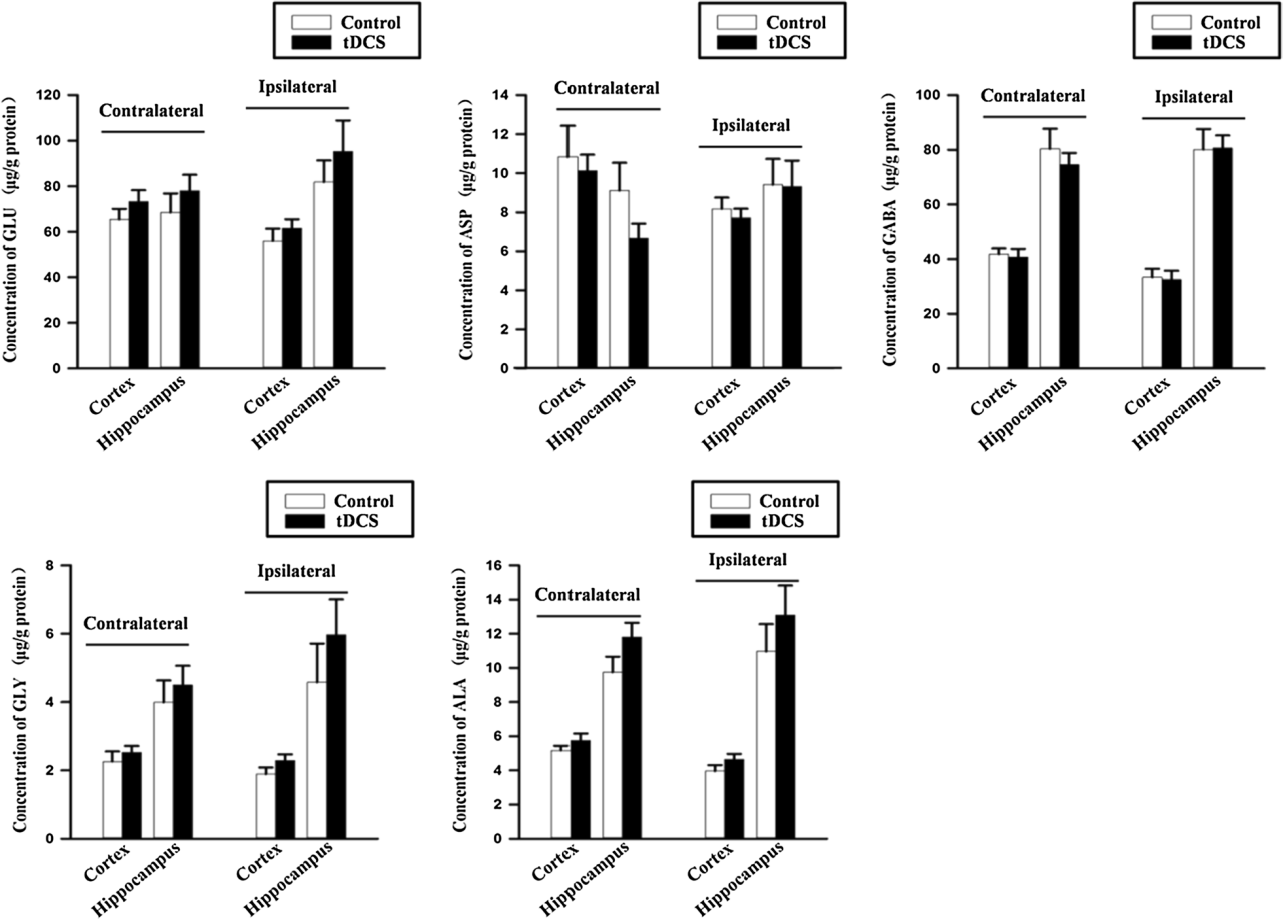
Neurotransmitter levels after cathodal tDCS. Data were presented as the mean ± SEM. N = 4 rats per group. No significant difference was observed between tDCS and control groups

Both excitatory (GLU and ASP) and inhibitory amino acids (GABA, GLY and ALA) play important roles in the modulation of neural activity. Therefore, in the present study we assessed all of these molecules using LC–MS chromatograms. Excitatory and inhibitory neurotransmitters in the tDCS group did not significantly differ in either the contralateral or ipsilateral hippocampus and cortex, compared with the control group (Fig. 5 and Additional file 10).

### 500 μA cathodal tDCS had no effects on cerebral temperature

**Fig. 6:**
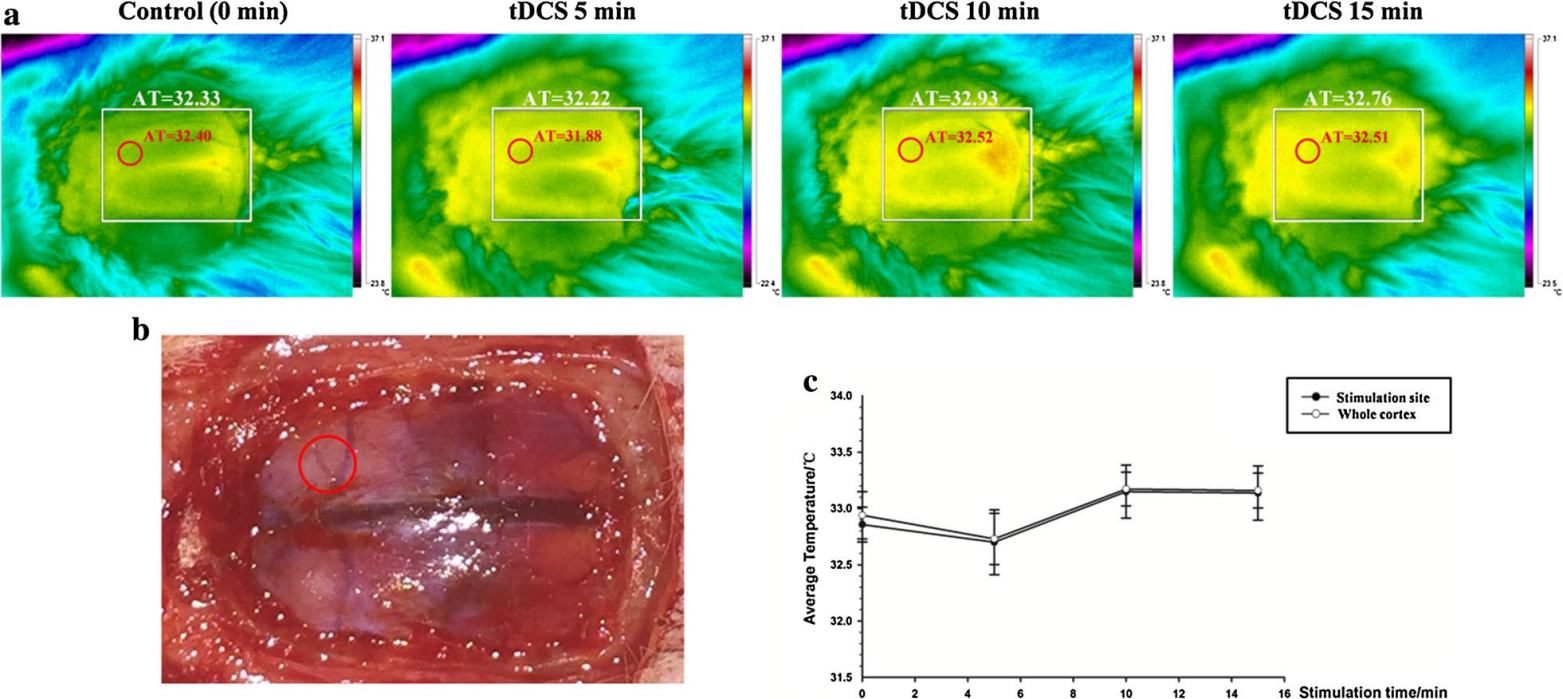
Changes in cerebral temperature during tDCS. Cerebral temperatures were visualized using a thermal imager and bundled software. **a** The red circle represents the cortex under the stimulating electrode, and the white square represents the entire cortex. **b** The stimulating electrode was located on the surface of the intact dura mater as depicted (red circle). **c** Cerebral temperature changes during tDCS treatment. Data were presented as the mean ± SEM. N = 4 rats per group. No significant differences were observed between the four observed time points. *AT* average temperature

In the present study, cerebral temperatures detected by thermal imaging after 15 min of cathodal tDCS exhibited no significant difference between the control and tDCS groups either when averaged over the entire cortex or when measured under the stimulation site. Furthermore, temperatures at the stimulation site did not significantly rise with increased stimulation time (Fig. 6a–c and Additional file 11).

## Discussion

tDCS is a powerful brain modulation tool validated by an increasing number of studies. tDCS treatment may lead to neuromodulation and lasting changes in cortical excitability. Although tDCS has demonstrated therapeutic potential, little is known about potential harmful effects of continuous weak direct current stimulation. Tissue heating (burning) through joule heat has been proposed as a probable mechanism for damage. Such heating is linearly dependent on charge density but is polarity-independent, assuming a linear relation between charge density (current density × time) and lesion size [12, 15]. We chose cathodal tDCS instead of anodal tDCS in the present study because cathodal tDCS has been shown to be effective in a range of neurological diseases [13, 14, 16, 17], and because the safety of 500 μA cathodal tDCS has remained controversial in rats [12–14].

As we know, protein denaturation usually occurs when the temperature of tissue is above 43 °C. Bikson et al. [15] reported that a tDCS charge density of 85,714 C/m^2^ would theoretically increase the temperature of brain tissue to 47.75 °C, in this case, probably it would lead to brain lesions. In the present study, a charge density of 128,571 C/m^2^ was applied; according to the theory of Bikson and colleagues [15], this density would induce much higher temperature and cause more obvious histopathological brain damage. However, no brain damage under the stimulation site was found, which were probably due to the complicated temperature self-regulating mechanism of living body, as well as the environmental temperature and the type of stimulating electrode. In addition, it was found that the current density in brain tissue (< 20 A/m^2^) and skull (40–60 A/m^2^) calculated by a computational model was much lower than that calculated with the formula (142.9 A/m^2^) [12]. We also found that the current density was different even in the same tissue but different spatial positions, which indicated that the charge density (current density × time) merely calculated by the formula in theory without taking these elements into consideration was unreasonable. In a previous study [12], tDCS was applied transcranially to the frontal cortex with 500 μA for 15 min, brain lesions were detected. However, in other studies [13, 14] and our study, no brain lesions were found after tDCS treatment on motor cortex with the same paremeters (500 μA for 15 min). Therefore, the position of stimulating electrode implanted may be a vital factor for safety evaluation. Besides, much higher current density distribution was found in skull instead of brain, which indicated that more joule heat generated in skull than that in brain tissue. Considering the previous reports that brain lesion occurred only in cortex under the skull where the stimulating electrode was implanted [12], we speculated that it was the joule heat produced in skull instead of in brain tissue itself caused the brain lesion.

Prior studies have reported that current injected into the brain during tDCS is diffused by the skull. Thus, the current is not focused on the brain surface [18, 19], which may lead to a weaker actual current. Other research has demonstrated that sponge electrodes can achieve better current on the scalp near the sponge edges [18, 20]. Additionally, a counter electrode with a large contact area placed on the ventral thorax creates an asymmetric current density with highest density directly beneath the epicranial electrode [21]. In this study, the similar electrode material to Liebetanz’s group [12] was used, but the contact area of our counter electrode placed on the ventral thorax was a little smaller (7 cm^2^) than Liebetanz’s group (10 cm^2^). Considering the inconsistent reulsts of biosafety of tDCS on brain between Liebetanz’s study and ours, the contact area of counter electrode should be considered. These findings indicated that the geometry and materials of tDCS play an important role in its biosafety and a recognized standard should be established for tDCS study.

Essential brain functions including motor function and learning and memory ability were not significantly affected by cathodal tDCS administration in present parameter (500 μA, 15 min, once per day, totally for 10 days). However, a robust meta-analysis indicated that anodal tDCS had significantly positive effects on cognitive and motor function in healthy older adults over a variety of experimental tasks [22]. Additionally, cathodal tDCS may exert positive effects on functional recovery after stroke; such recovery involves neurogenesis, oligodendrocyte precursor recruitment, and microglial polarization [13]. The inconsistency of the present results with the literature may be due to differences in the modulatory effect of tDCS, selected parameters, position of the implanted stimulating electrode, or even the composition of study samples (e.g., mice, rats or humans).

This study assessed not only the effect of cathodal tDCS on the brain but also on animals’ physiological health. Such health-related outcomes have been largely ignored in previous research. Although it is well understood that current from tDCS flows through the entire body, the effects of this current have only been evaluated in the brain; whether the current is beneficial or harmful to other organs has remained unknown. Therefore, the current study assessed the morphology of major organs and selected physiological indexes. No morphological effects were observed in the brain, heart or other major organs. Similarly, no obvious change was found in hematology and serum biochemical markers. These results suggested that the cathodal tDCS protocol used in the current study does not affect the health of animals.

Additionally, levels of neurotransmitters and Nissl bodies in the brain were determined to evaluate potential brain damage after tDCS treatment. Neither the level of excitatory and inhibitory neurotransmitters, nor the Nissl bodies in cortex and hippocampus were affected by cathodal tDCS, which was consistent with the behavioral results. However, Peruzzotti-Jametti et al. reported decreases in cortical GLU and ALA after cathodal tDCS treatment in mice [17], indicating that tDCS may promote efficient conversion of GLU into glutamine [23]. Inconsistencies between the results of our study and the previous report may be due to the differences of the experimental animals, tDCS duration and tDCS site.

## Conclusions

In conclusion, our results demonstrated that 500 μA cathodal tDCS had no effects on the behavior of adult male rats, including locomotor activity, athletic endurance and spatial learning and memory. Moreover, hematology indexes, serum biochemical markers and brain neurotransmitters were not altered by tDCS treatment. Furthermore, tDCS did not affect the major organs’ morphology and cerebral temperature. The data suggested that 500 μA cathodal tDCS performed in present study was safe for rodents.

## Methods

### Animals and groups

Male Sprague–Dawley rats (260–280 g) purchased from the Laboratory Animal Center of the Fourth Military Medical University (Xi’an, China) were housed in a temperature-controlled room in plastic cages (3 animals per cage) with free access to food and water at 22–25 °C on a 12 h light/dark cycle. We selected 24 animals that were capable of running continuously on the rotarod for a minimum of 300 s at 4 rpm. These animals were randomly divided into two groups: a cathodal tDCS group (tDCS, n = 12) and control group (control, n = 12). All experiments reported in this study were conducted according to an experimental protocol approved by the Animal Use and Care Committee for Research and Education of Fourth Military Medical University (NO. 20170607). After experiments, animals were deeply anesthetized by intraperitoneal injection of pentobarbital (60 mg/kg) to minimize the pain of the animals.

### tDCS administration

Three days before the administration of tDCS, epicranial electrode implantation was carried out in rats under 1% pentobarbital sodium anesthesia (45 mg/kg i.p.). After removal of the scalp and underlying tissues, an epicranial electrode with a defined contact area of 3.5 mm^2^ was mounted on the intact skull at the coordinates bregma AP + 2.0 mm, ML + 2.0 mm using glass ionomer dental cement (Ketac Cem, ESPE Dental AG, Seefeld, Germany). This electrode remained in place for the duration of the experiment. The counter electrode, a large conventional rubber plate electrode (7 cm^2^, Ai Kang Technology, China), was placed onto the ventral thorax of the unrestrained animal using a corset as previously described [12]. Animals received 5 consecutive days of tDCS (500 μA, 15 min, once per day) followed by a tDCS-free interval of 2 days and 5 additional days of stimulation. Thus, tDCS administration comprised 10 days in total; this duration has been frequently adopted in studies of therapeutic tDCS effects on disease states [13, 14].

Prior to tDCS, the stimulation electrode was filled with saline solution (0.9% NaCl) to reduce the skull impedance [24]. To avoid stimulation break effects, the current intensity was automatically ramped up and down over a 10 s period instead of being switched on and off abruptly [25]. tDCS was applied continuously for 15 min at a current strength of 500 μA using a constant current stimulator (PMP18-3TR, Kikusui, Japan), resulting in a charge density of 128,571 C/m^2^. Notably, animals were maintained in an awake state during tDCS to avoid possible interactions between tDCS and anesthetic. Prior research has reported interactions of tDCS with benzodiazepines and another antidepressant drugs [26]. Furthermore, stimulation in awake rats may mimic the clinical application of tDCS.

### Computational model

In this study, a 3D rat model obtained from Brooks Fourth Military Laboratory (BAFL) was used to calculate the current density distributions in rat brain during tDCS treatment. The model has 36 different tissues with voxel size 0.5 mm × 1 mm × 0.5 mm. The electrical properties of head tissues were modeled using the 4-Cole–Cole method and obtained by fitting to experimental measurements [27]. The currents distributions in rat model was calculated by the impedance method [28]. In brief, the rat model was described by using a uniform 3D Cartesian grid and was composed of small cubic voxels. There are nearly seven million voxels in the computational space. Assuming that, in each voxel, the electric conductivities are isotropic and constant in all directions, the model was represented as a 3D network of impedances. Kirchhoff voltage law applied in this network generates a system of equations for the loop currents. These loops currents were driven by the injected current from the tDCS electrodes. The net currents and current density within the rat model are then calculated from these loop currents, and the electric field are in turn calculated using Ohm’s Law.

### Behavioral assessment

#### Open field test

Immediately after tDCS administration, the open field test (OFT) was conducted blindly to evaluate locomotor activity [29]. Rats were gently placed at the center of the open field device (100 cm × 100 cm × 40 cm) with their backs towards the investigator. Each rat was allowed to explore freely for 5 min. Movement traces and total distance moved were recorded and automatically analyzed by the Noldus Ethovision XT 5 video tracking system. Prior to each testing session, animals were habituated to the testing environment for at least 30 min. The device was wiped with 70% ethanol and disposable absorbent paper before each rat was tested. During testing, light and sound cues were controlled to prevent these cues from influencing the results. A baseline measure of total distance traveled was acquired 2 days before tDCS administration (BEFORE). The OFT was conducted three times during the tDCS administration period: once each in the early phase (ERLY, immediately after 2 days of tDCS), middle phase (MID, 24 h after the fifth day of tDCS) and post-stimulation phase (POST, immediately after the tenth day of tDCS).

#### Rotarod test

Immediately after OFT detection, about 1.5 h after tDCS administration, athletic endurance was evaluated with a rotarod treadmill for rats (Ugo Basile, Italy), under the accelerating rotor mode (10 speeds from 4 to 40 rpm for 5 min) as previously described [30]. The duration from the time the animal mounted the rod to the time it fell off was recorded as the performance time. Performance on the rotarod test was measured 5 times per day with a minimum interval between two trials of 10 min to ensure energy recovery. Animals were trained for 5 trials per day at the speed of 4 rpm for 3 days before tDCS administration, and baseline performance (BEFORE) was recorded on the last training day. Like the OFT, the rotarod test was performed in the ERLY, MID and POST phases.

#### Morris water maze test

After rotarod test, animals were allowed to rest for 2 h, and then about 5 h after tDCS administration, the Morris water maze (MWM) test was administered under dim white light in a black plastic pool 150 cm in diameter. Rats were trained for 5 days consecutively. Each animal received 4 trials per day from different randomly-selected quadrants with target location remain constant; the latency to reach the platform was recorded [31]. Rats were allowed to swim until they reached the platform or until 60 s had elapsed. Rats who were unable to locate the MWM platform within the allotted time were led to the platform by the experimenter. Rats were allowed to remain on the platform for 15 s and then removed from the pool. The interval between trials was at least 10 min, and animals were placed in holding cages in front of an air heater. To assess reference memory at the end of learning, a probe trial was given 24 h after the last acquisition day from the quadrant contralateral to target quadrant. During the probe trial the platform was removed, and the duration in the target quadrant was measured; this observation served as the baseline (BEFORE). We note that 24 h before the probe trial in ERLY, MID and POST phases, an extra spatial acquisition training was performed. Prior to performance, the animals were habituated to the testing environment for at least 30 min and the water temperature was equilibrated to room temperature (23 °C).

### Hematological analysis and serum biochemical marker assay

After 10 days of tDCS administration, animals were deeply anesthetized by intraperitoneal injection of pentobarbital (60 mg/kg), and blood samples were taken from the left ventricle of the heart. Samples of 1 ml were collected in EDTA-K2 vacuum tubes (Boyi Medical Equipment Company, Shandong, China). Hematological analysis was performed with an automated hematology analyzer (XE-2100, Sysmex, Tokyo, Japan) and the number of white blood cells (WBC) and platelets (PLT), the percentage of lymphocytes (LYM) and neutrophile granulocytes (GRA), and the concentration of hemoglobin (HGB) were detected. Additionally, 3 ml of serum was collected from blood after centrifugation at 3000 rpm for 10 min at 4 °C. Serum biochemical markers including markers of liver and kidney function, such as alanine aminotransferase (ALT), aspartate transaminase (AST), alkaline phosphatase (ALP), total bilirubin (TBILI), albumin (ALB) serum creatinine (Scr), blood urea nitrogen (BUN) and cystatin C (Cys C), were detected by commercial kits and a multifunctional biochemistry analyzer (AU600; Olympus, Tokyo, Japan) according to the manufacturer’s instructions.

### HE staining and Nissl’s staining

After the collection of blood samples, animals were immediately transcardially perfused with 200 ml of 5 mM sodium phosphate-buffered 0.9% (w/v) saline (PBS, pH 7.2–7.4) and 4% paraformaldehyde in a phosphate buffer (0.1 M, pH 7.4). The major organs, including the brain, heart, lung, liver, kidney and spleen, were embedded in paraffin. Continuous 4 μm thick horizontal slices were obtained. For hematoxylin and eosin (HE) staining, the sections were dipped in hematoxylin for 3 min, washed in running tap water until the sections appeared blue to the naked eyes, and destained in hydrochloric acid alcohol for several seconds. The sections were washed again in running water for 10 min and dipped in eosin for 15 s prior to washing again for 5 min. They were subsequently dehydrated in an alcohol gradient, cleared in xylene, and coverslipped. For Nissl’s staining, the sections were dipped in 1% toluidine blue for 30 min at 45 °C, and differentiated in 75% alcohol for seconds, then rinsed quickly in distilled water. Digital images were acquired using an inverted microscope (TE2000-S, Nikon, Tokyo, Japan).

### Liquid chromatograph mass spectrometer (LC–MS)

Animals were anesthetized and sacrificed as previously described. The hippocampus and surrounding cortex ipsilateral to the tDCS site as well as their contralateral homologues were immediately separated and frozen to avoid protein degradation. Samples were subsequently weighed and homogenized with 0.4 ml of a solution of 1% formic acid prepared in cold (− 20 °C) acetonitrile using a homogenate device (Leica, Heidelberg, Germany). Two hundred microliters of the supernatant were collected after centrifugation at 12,000 rpm for 20 min at 4 °C. Levels of neurotransmitters such as glutamic acid (GLU), aspartic acid (ASP), γ-aminobutyric acid (GABA), glycine (GLY) and alanine (ALA) were determined using LC–MS equipment (QqQ-6410B, Agilent Technologies, China) as reported [32]. This equipment was provided by the Centers for Disease Control and Prevention of Xi ‘an.

### Thermal imaging

Cerebral temperature changes during tDCS are an important safety factor associated with risk for brain damage [15]. A thermal imager (Ti400, FLUKE, USA) was used to monitor and visualize temperature changes in the brain during the 15 min stimulation period of 500 μA cathodal tDCS. The bundled software (SmartView 3.14, FLUKE, USA) was used to measure temperature. The brain temperature detected before tDCS was considered the control, and average temperatures for cortex under the stimulation site as well as for the entire cortex were recorded every 5 min during the stimulation period. Each animal received 3 trials at each test point. Since the thermal imager used in this study could only detect the surface temperature, in order to get accurate brain temperature changes after tDCS stimulation, the electrode was not fixed on the intact skull with dental cement as before, instead, it was placed on the surface of the intact dura mater after removal of the scalp and skull by using a stereotaxic apparatus (RWD life Science, Shengzhen, China) and then the surface temperature of stimulating site and the whole brain was measured.

### Statistical analysis

All data were collected and analyzed by researchers blinded to the surgery and reagents that were used. Repeated measures analysis of variance (ANOVA) were conducted on data from the behavioral experiments and on cerebral temperature values. Hematology results, serum biochemical markers, and neurotransmitter levels were analyzed using a one-way ANOVA. All data were presented as the mean ± standard error of mean (SEM), and all statistical analysis were performed using SPSS version 20.0 (SPSS Inc., Chicago, IL, USA). *p*-values less than 0.05 were considered statistically significant.

## Additional files

**Additional file 1.** Induced current density (A/m^2^) along test lines in rat brain tissues at coronal plane of y = 52 mm.

**Additional file 2.** Total distance traveled in the OFT.

**Additional file 3.** Retention time on the rotarod.

**Additional file 4.** Latency to the platform on the spatial acquisition test.

**Additional file 5.** Durations in target quadrant on probe trials.

**Additional file 6.** The results of hematology.

**Additional file 7.** Levels of serum biochemical markers for liver function.

**Additional file 8.** Levels of serum biochemical markers for kidney function.

**Additional file 9.** Quantitative analysis of Nissl bodies.

**Additional file 10.** The results of Neurotransmitter levels.

**Additional file 11.** Cerebral temperature changes during tDCS treatment.

## Data Availability

Raw data has been provided as additional file.

## Abbreviations

tDCS: transcranial direct current stimulation
OFT: open field test
MWM: Morris water maze
WBC: white blood cells
PLT: platelets
LYM: lymphocytes
GRA: granulocytes
HGB: hemoglobin
ALT: alanine aminotransferase
AST: aspartate transaminase
ALP: alkaline phosphatase
TBILI: total bilirubin
ALB: albumin
Scr: serum creatinine
BUN: blood urea nitrogen
Cys C: cystatin C
HE: hematoxylin and eosin
PBS: phosphate-buffered saline
LC–MS: liquid chromatograph mass spectrometer
GLU: glutamic acid
ASP: aspartic acid
GABA: γ-aminobutyric acid
GLY: glycine
ALA: alanine
ANOVA: analysis of variance
SEM: standard error of mean
AT: average temperature
