# Supplementary material for: A safety study of 500 μA cathodal transcranial direct current stimulation in rat
Source: BMC Neurosci. 2019 Aug 6;20:40. doi: 10.1186/s12868-019-0523-7 (PMC6683582; doi:10.1186/s12868-019-0523-7)
Supplement: Supplementary file 1 — Additional file 1. Induced current density (A/m2) along test lines in rat brain tissues at coronal plane of y = 52 mm. [file 12868_2019_523_MOESM1_ESM.docx]

**Additional file 1** Induced current density (A/m^2^) along test lines in rat brain tissues at coronal plane of y=52 mm.

|  | **GM** | **GM** | **GM** | **GM** | **GM** |
| --- | --- | --- | --- | --- | --- |
| Test Line-1 | 6.836 | 6.096 | 6.806 | 6.351 | 6.369 |
| Test Line-2 | 5.984 | 5.986 | 5.972 | 5.945 | 5.868 |
| Test Line-3 | 4.956 | 4.919 | 4.787 | 4.585 | 4.351 |


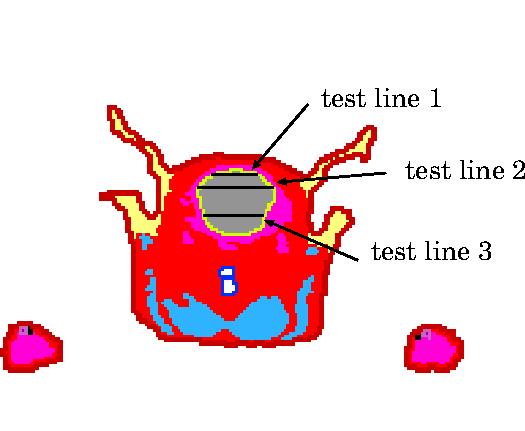


**Test line-1**: 7mm depth from the vertex of the scalp.

**Test line-2**: 15mm depth from the vertex of the scalp.

**Test line-3**: 22 mm depth from the vertex of the scalp.
