# Supplementary material for: A safety study of 500 μA cathodal transcranial direct current stimulation in rat
Source: BMC Neurosci. 2019 Aug 6;20:40. doi: 10.1186/s12868-019-0523-7 (PMC6683582; doi:10.1186/s12868-019-0523-7)
Supplement: Supplementary file 2 — Additional file 2. Total distance traveled in the OFT. [file 12868_2019_523_MOESM2_ESM.docx]

**Additional file 2** Total distance traveled in the OFT.

| **Group** | **ID** | **BEFORE** | **ERLY** | **MID** | **POST** |
| --- | --- | --- | --- | --- | --- |
| Control | 1 | 2499.79 | 3086.93 | 2806.06 | 2509.86 |
| Control | 2 | 3047.01 | 3650.13 | 3158.34 | 2556.93 |
| Control | 4 | 3861.09 | 2780.80 | 2757.60 | 3409.39 |
| Control | 8 | 3553.03 | 2302.43 | 2803.81 | 3322.33 |
| Control | 10 | 3511.43 | 3369.13 | 3475.90 | 3428.66 |
| Control | 11 | 3390.81 | 2500.64 | 3234.61 | 2660.68 |
| tDCS | 3 | 3070.12 | 2606.06 | 3189.76 | 2536.63 |
| tDCS | 5 | 3430.34 | 3321.06 | 2807.01 | 3464.60 |
| tDCS | 6 | 4518.98 | 3777.34 | 4470.53 | 3993.62 |
| tDCS | 7 | 3530.98 | 3224.00 | 3826.54 | 2986.46 |
| tDCS | 9 | 2042.49 | 2417.96 | 3071.13 | 3483.35 |
| tDCS | 12 | 4151.30 | 2884.66 | 2866.33 | 2405.56 |
