# Supplementary material for: A safety study of 500 μA cathodal transcranial direct current stimulation in rat
Source: BMC Neurosci. 2019 Aug 6;20:40. doi: 10.1186/s12868-019-0523-7 (PMC6683582; doi:10.1186/s12868-019-0523-7)
Supplement: Supplementary file 3 — Additional file 3. Retention time on the rotarod. [file 12868_2019_523_MOESM3_ESM.docx]

**Additional file 3** Retention time on the rotarod.

| **Group** | **ID** | **BEFORE** | **ERLY** | **MID** | **POST** |
| --- | --- | --- | --- | --- | --- |
| Control | 1 | 136.00 | 114.10 | 146.33 | 149.37 |
| Control | 2 | 163.00 | 153.90 | 132.00 | 127.85 |
| Control | 4 | 157.10 | 138.90 | 108.70 | 147.23 |
| Control | 8 | 103.50 | 115.40 | 111.77 | 110.67 |
| Control | 10 | 128.80 | 94.60 | 98.03 | 125.90 |
| Control | 11 | 129.00 | 123.40 | 119.40 | 127.89 |
| tDCS | 3 | 138.00 | 116.80 | 119.93 | 134.00 |
| tDCS | 5 | 124.00 | 162.40 | 119.63 | 141.53 |
| tDCS | 6 | 143.10 | 138.80 | 118.97 | 118.80 |
| tDCS | 7 | 135.00 | 95.80 | 126.00 | 118.97 |
| tDCS | 9 | 123.00 | 104.50 | 109.97 | 111.63 |
| tDCS | 12 | 130.10 | 123.60 | 118.90 | 128.00 |
