# Supplementary material for: A safety study of 500 μA cathodal transcranial direct current stimulation in rat
Source: BMC Neurosci. 2019 Aug 6;20:40. doi: 10.1186/s12868-019-0523-7 (PMC6683582; doi:10.1186/s12868-019-0523-7)
Supplement: Supplementary file 4 — Additional file 4. Latency to the platform on the spatial acquisition test. [file 12868_2019_523_MOESM4_ESM.docx]

**Additional file 4** Latency to the platform on the spatial acquisition test.

| **Group** | **ID** | **-5** | **-4** | **-3** | **-2** | **-1** | **2** | **5** | **11** |
| --- | --- | --- | --- | --- | --- | --- | --- | --- | --- |
| Control | 1 | 60.00 | 60.00 | 33.70 | 19.93 | 26.75 | 33.50 | 14.00 | 24.75 |
| Control | 2 | 60.00 | 28.00 | 51.30 | 52.70 | 46.00 | 37.00 | 21.75 | 40.75 |
| Control | 4 | 60.00 | 51.00 | 47.23 | 40.33 | 44.25 | 26.75 | 24.75 | 25.50 |
| Control | 8 | 59.63 | 39.00 | 40.95 | 29.03 | 29.75 | 30.75 | 13.50 | 23.50 |
| Control | 10 | 48.48 | 45.00 | 23.33 | 22.53 | 19.00 | 26.00 | 34.25 | 29.75 |
| Control | 11 | 60.00 | 30.00 | 34.28 | 39.13 | 41.75 | 19.75 | 15.75 | 22.75 |
| tDCS | 3 | 60.00 | 48.00 | 28.17 | 19.85 | 37.25 | 23.75 | 17.12 | 48.00 |
| tDCS | 5 | 60.00 | 60.00 | 38.38 | 28.25 | 25.50 | 42.25 | 17.25 | 28.25 |
| tDCS | 6 | 60.00 | 45.00 | 27.05 | 40.23 | 48.25 | 19.00 | 36.00 | 20.50 |
| tDCS | 7 | 60.00 | 55.00 | 35.85 | 33.60 | 31.50 | 31.50 | 28.00 | 44.00 |
| tDCS | 9 | 60.00 | 45.00 | 41.03 | 33.33 | 23.00 | 28.75 | 23.50 | 35.50 |
| tDCS | 12 | 60.00 | 44.00 | 38.73 | 29.95 | 17.50 | 17.25 | 12.00 | 14.25 |
