# Supplementary material for: A safety study of 500 μA cathodal transcranial direct current stimulation in rat
Source: BMC Neurosci. 2019 Aug 6;20:40. doi: 10.1186/s12868-019-0523-7 (PMC6683582; doi:10.1186/s12868-019-0523-7)
Supplement: Supplementary file 5 — Additional file 5. Durations in target quadrant on probe trials. [file 12868_2019_523_MOESM5_ESM.docx]

**Additional file 5** Durations in target quadrant on probe trials.

| **Group** | **ID** | **BEFORE** | **ERLY** | **MID** | **POST** |
| --- | --- | --- | --- | --- | --- |
| Control | 1 | 10.12 | 15.00 | 20.88 | 23.16 |
| Control | 2 | 7.44 | 10.00 | 23.40 | 26.88 |
| Control | 4 | 26.44 | 22.00 | 21.76 | 22.00 |
| Control | 8 | 23.24 | 23.00 | 24.32 | 13.04 |
| Control | 10 | 10.60 | 18.00 | 40.44 | 20.28 |
| Control | 11 | 21.16 | 19.00 | 32.00 | 28.24 |
| tDCS | 3 | 20.44 | 19.00 | 44.20 | 22.00 |
| tDCS | 5 | 16.88 | 19.00 | 28.64 | 19.24 |
| tDCS | 6 | 12.28 | 17.00 | 28.28 | 20.40 |
| tDCS | 7 | 7.04 | 20.00 | 27.36 | 23.44 |
| tDCS | 9 | 12.20 | 22.00 | 38.44 | 20.72 |
| tDCS | 12 | 27.56 | 20.00 | 24.28 | 20.68 |
