# Supplementary material for: A safety study of 500 μA cathodal transcranial direct current stimulation in rat
Source: BMC Neurosci. 2019 Aug 6;20:40. doi: 10.1186/s12868-019-0523-7 (PMC6683582; doi:10.1186/s12868-019-0523-7)
Supplement: Supplementary file 6 — Additional file 6. The results of hematology. [file 12868_2019_523_MOESM6_ESM.docx]

**Additional file 6** The results of hematology.

| **Group** | **ID** | **WBC (10^9^/L)** | **HGB (g/L)** | **PLT (10^9^/L)** | **LYM (%)** | **GRA (%)** |
| --- | --- | --- | --- | --- | --- | --- |
| Control | 1 | 6.23 | 127.00 | 1012.00 | 0.20 | 0.75 |
| Control | 2 | 10.89 | 119.00 | 812.00 | 0.30 | 0.56 |
| Control | 4 | 5.35 | 122.00 | 1015.00 | 0.32 | 0.55 |
| Control | 8 | 3.42 | 131.00 | 1041.00 | 0.22 | 0.74 |
| Control | 10 | 6.12 | 129.00 | 637.00 | 0.36 | 0.57 |
| Control | 11 | 6.40 | 125.60 | 903.40 | 0.28 | 0.63 |
| tDCS | 3 | 10.73 | 123.00 | 463.00 | 0.18 | 0.53 |
| tDCS | 5 | 6.87 | 113.00 | 1233.00 | 0.17 | 0.75 |
| tDCS | 6 | 6.29 | 128.00 | 917.00 | 0.27 | 0.65 |
| tDCS | 7 | 7.94 | 120.40 | 751.80 | 0.24 | 0.75 |
| tDCS | 9 | 7.27 | 117.00 | 728.00 | 0.28 | 0.66 |
| tDCS | 12 | 8.58 | 121.00 | 418.00 | 0.30 | 0.50 |
