# Supplementary material for: A safety study of 500 μA cathodal transcranial direct current stimulation in rat
Source: BMC Neurosci. 2019 Aug 6;20:40. doi: 10.1186/s12868-019-0523-7 (PMC6683582; doi:10.1186/s12868-019-0523-7)
Supplement: Supplementary file 7 — Additional file 7. Levels of serum biochemical markers for liver function. [file 12868_2019_523_MOESM7_ESM.docx]

**Additional file 7** Levels of serum biochemical markers for liver function.

| **Group** | **ID** | **ALT** | **AST** | **ALB** | **TBILI** | **ALP** |
| --- | --- | --- | --- | --- | --- | --- |
| Control | 1 | 40.00 | 125.00 | 21.90 | 0.20 | 203.00 |
| Control | 2 | 37.00 | 142.00 | 21.20 | 0.30 | 237.00 |
| Control | 4 | 36.00 | 106.00 | 21.10 | 0.60 | 321.00 |
| Control | 8 | 32.00 | 84.00 | 23.60 | 0.50 | 458.00 |
| Control | 10 | 35.60 | 143.00 | 21.20 | 0.30 | 289.80 |
| Control | 11 | 33.00 | 153.00 | 20.60 | 0.40 | 230.00 |
| tDCS | 3 | 29.40 | 98.60 | 20.28 | 0.30 | 302.00 |
| tDCS | 5 | 25.00 | 81.00 | 18.40 | 0.40 | 297.00 |
| tDCS | 6 | 32.00 | 89.00 | 22.70 | 0.80 | 267.00 |
| tDCS | 7 | 30.00 | 133.00 | 21.70 | 0.80 | 438.00 |
| tDCS | 9 | 38.00 | 139.00 | 20.80 | 0.10 | 256.00 |
| tDCS | 12 | 22.00 | 111.00 | 17.80 | 0.48 | 252.00 |
