# Supplementary material for: A safety study of 500 μA cathodal transcranial direct current stimulation in rat
Source: BMC Neurosci. 2019 Aug 6;20:40. doi: 10.1186/s12868-019-0523-7 (PMC6683582; doi:10.1186/s12868-019-0523-7)
Supplement: Supplementary file 8 — Additional file 8. Levels of serum biochemical markers for kidney function. [file 12868_2019_523_MOESM8_ESM.docx]

**Additional file 8** Levels of serum biochemical markers for kidney function.

| **Group** | **ID** | **CysC** | **BUN** | **Scr** |
| --- | --- | --- | --- | --- |
| Control | 1 | 0.03 | 8.60 | 48.00 |
| Control | 2 | 0.03 | 7.19 | 53.00 |
| Control | 4 | 0.03 | 6.96 | 51.00 |
| Control | 8 | 0.05 | 6.68 | 54.00 |
| Control | 10 | 0.05 | 6.29 | 50.00 |
| Control | 11 | 0.05 | 6.35 | 49.00 |
| tDCS | 3 | 0.03 | 7.43 | 51.00 |
| tDCS | 5 | 0.03 | 7.04 | 55.00 |
| tDCS | 6 | 0.02 | 7.89 | 57.00 |
| tDCS | 7 | 0.05 | 6.44 | 51.00 |
| tDCS | 9 | 0.05 | 8.07 | 53.00 |
| tDCS | 12 | 0.05 | 7.74 | 51.00 |
