# Supplementary material for: A safety study of 500 μA cathodal transcranial direct current stimulation in rat
Source: BMC Neurosci. 2019 Aug 6;20:40. doi: 10.1186/s12868-019-0523-7 (PMC6683582; doi:10.1186/s12868-019-0523-7)
Supplement: Supplementary file 9 — Additional file 9. Quantitative analysis of Nissl bodies. [file 12868_2019_523_MOESM9_ESM.docx]

**Additional file 9** Quantitative analysis of Nissl bodies (Chose 3 horizon randomly)

| **Group** | **Density of Nissl Bodies in Hipppcampus per Horizon** | **Density of Nissl Bodies in Hipppcampus per Horizon** |
| --- | --- | --- |
| Control | 459 | 74 |
| Control | 466 | 80 |
| Control | 483 | 81 |
| tDCS | 479 | 89 |
| tDCS | 486 | 73 |
| tDCS | 453 | 77 |
