# Supplementary material for: A safety study of 500 μA cathodal transcranial direct current stimulation in rat
Source: BMC Neurosci. 2019 Aug 6;20:40. doi: 10.1186/s12868-019-0523-7 (PMC6683582; doi:10.1186/s12868-019-0523-7)
Supplement: Supplementary file 10 — Additional file 10. The results of Neurotransmitter levels. [file 12868_2019_523_MOESM10_ESM.docx]

**Additional file 10** The results of Neurotransmitter levels.

| **Group** | **Brain regions** | **GLU** | **GABA** | **GLY** | **ASP** | **ALA** |
| --- | --- | --- | --- | --- | --- | --- |
| Control | left cortex | 72.43 | 45.05 | 1.93 | 15.43 | 5.75 |
| Control | left cortex | 62.83 | 40.14 | 1.64 | 9 | 5.05 |
| Control | left cortex | 73.11 | 45.33 | 2.51 | 10.59 | 5.38 |
| Control | left cortex | 53.07 | 36.55 | 2.95 | 8.31 | 4.5 |
| tDCS | left cortex | 73.18 | 42.32 | 2.46 | 10.18 | 6.13 |
| tDCS | left cortex | 78.31 | 43.94 | 2.71 | 12.14 | 6.71 |
| tDCS | left cortex | 59.47 | 32.02 | 2.01 | 8.11 | 5.03 |
| tDCS | left cortex | 82.25 | 44.79 | 2.9 | 10.06 | 5.16 |
| Control | left hippocampus | 80.55 | 77.08 | 3.09 | 12.15 | 10.45 |
| Control | left hippocampus | 56.65 | 60.84 | 2.72 | 6.02 | 7.02 |
| Control | left hippocampus | 85 | 93.91 | 4.82 | 7.47 | 10.74 |
| Control | left hippocampus | 51.54 | 89.4 | 5.34 | 10.79 | 10.77 |
| tDCS | left hippocampus | 58.18 | 63.83 | 3.01 | 4.45 | 10.11 |
| tDCS | left hippocampus | 82.68 | 80.64 | 5.68 | 7.3 | 13.95 |
| tDCS | left hippocampus | 79.64 | 72.04 | 4.97 | 7.17 | 12.13 |
| tDCS | left hippocampus | 91.41 | 81.99 | 4.32 | 7.75 | 11.07 |
| Control | right cortex | 55.32 | 34.85 | 1.85 | 8.66 | 3.89 |
| Control | right cortex | 52.96 | 30.45 | 1.51 | 7.52 | 3.55 |
| Control | right cortex | 70.81 | 41.26 | 2.42 | 9.55 | 4.97 |
| Control | right cortex | 44.88 | 26.83 | 1.79 | 6.98 | 3.39 |
| tDCS | right cortex | 72.9 | 42.01 | 2.6 | 9.07 | 5.39 |
| tDCS | right cortex | 55.21 | 28.82 | 2.32 | 7.49 | 4.12 |
| tDCS | right cortex | 59.79 | 27.7 | 2.45 | 6.9 | 4.96 |
| tDCS | right cortex | 58.42 | 31.31 | 1.8 | 7.38 | 4.1 |
| Control | right hippocampus | 70.06 | 74.92 | 3.11 | 11.13 | 10.62 |
| Control | right hippocampus | 82.9 | 91.08 | 3.49 | 8.71 | 11.04 |
| Control | right hippocampus | 66.04 | 60.92 | 3.75 | 6.02 | 7.16 |
| Control | right hippocampus | 108.18 | 93.24 | 7.96 | 11.8 | 15 |
| tDCS | right hippocampus | 75.7 | 71.75 | 4.7 | 8.38 | 12.11 |
| tDCS | right hippocampus | 106.74 | 92.45 | 7.49 | 11.26 | 16.04 |
| tDCS | right hippocampus | 128.2 | 83.28 | 7.95 | 11.63 | 15.57 |
| tDCS | right hippocampus | 70.33 | 74.85 | 3.76 | 6.02 | 8.64 |
