# Supplementary material for: A safety study of 500 μA cathodal transcranial direct current stimulation in rat
Source: BMC Neurosci. 2019 Aug 6;20:40. doi: 10.1186/s12868-019-0523-7 (PMC6683582; doi:10.1186/s12868-019-0523-7)
Supplement: Supplementary file 11 — Additional file 11. Cerebral temperature changes during tDCS treatment. [file 12868_2019_523_MOESM11_ESM.docx]

**Additional file 11** Cerebral temperature changes during tDCS treatment.

| **Time points** | **Cerebral temperature under stimulation site** | **Cerebral temperature of whole cortex** |
| --- | --- | --- |
| 0 | 33.08 | 33.29 |
| 0 | 32.40 | 32.33 |
| 0 | 33.00 | 33.09 |
| 0 | 32.95 | 33.05 |
| 5 | 33.23 | 33.12 |
| 5 | 31.88 | 32.22 |
| 5 | 32.83 | 32.47 |
| 5 | 32.86 | 33.11 |
| 10 | 33.25 | 33.17 |
| 10 | 32.52 | 32.92 |
| 10 | 33.18 | 33.59 |
| 10 | 33.65 | 33.01 |
| 15 | 33.12 | 33.08 |
| 15 | 32.51 | 32.76 |
| 15 | 33.24 | 33.47 |
| 15 | 33.68 | 33.33 |
